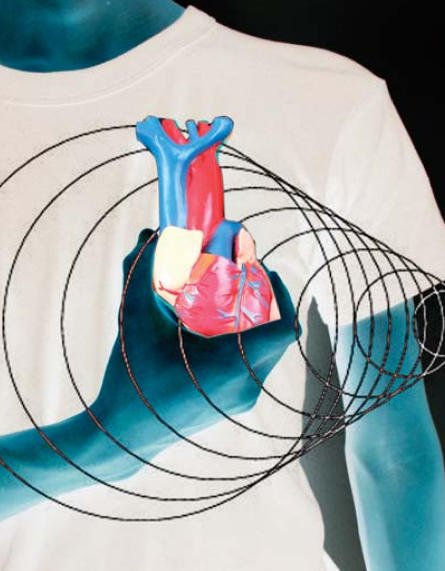# Noise Pollution: The Sound Behind Heart Effects

**DOI:** 10.1289/ehp.115-a536b

**Published:** 2007-11

**Authors:** M. Nathaniel Mead

More than 15 million Americans currently have some form of coronary heart disease (CHD), which involves a narrowing of the small blood vessels that supply blood and oxygen to the heart. Risk factors for CHD include diabetes, high blood pressure, altered blood lipids, obesity, smoking, menopause, and inactivity. To this list we can now add noise, thanks to a recent study and assessment of the evidence by the WHO Noise Environmental Burden on Disease working group. The findings, first presented at the Internoise 2007 conference in August 2007, will be published in December.

“The new data indicate that noise pollution is causing more deaths from heart disease than was previously thought,” says working group member Deepak Prasher, a professor of audiology at University College in London—perhaps hundreds of thousands around the world. “Until now, the burden of disease related to the general population’s exposure to environmental noise has rarely been estimated in nonoccupational settings at the international level.”

The separate noise-related working group first convened in 2003 and began sifting through data from studies in European countries to derive preliminary estimates of the impact of noise on the entire population of Europe. They then sought to separate the noise-related health effects from those of traffic-related air pollution and other confounding factors such as physical inactivity and smoking. In 2007, the group published *Quantifying Burden of Disease from Environmental Noise*, their preliminary findings on the health-related effects of noise for Europeans. Their conclusion: about 2% of Europeans suffer severely disturbed sleep, and 15% suffer severe annoyance due to environmental noise, defined as community noise emitted from sources such as road traffic, trains, and aircraft.

According to the new figures, long-term exposure to traffic noise may account for approximately 3% of CHD deaths (or about 210,000 deaths) in Europe each year. To obtain the new estimates, the working group compared households with abnormally high noise exposure with those with quieter homes. They also reviewed epidemiologic data on heart disease and hypertension, and then integrated these data into maps showing European cities with different levels of environmental noise.

The noise threshold for cardiovascular problems was determined to be a chronic nighttime exposure of at least 50 A-weighted decibels, the noise level of light traffic. Daytime noise exposures also correlated with health problems, but the risk tended to increase during the nighttime hours. “Many people become habituated to noise over time,” says Prasher. “The biological effects are imperceptible, so that even as you become accustomed to the noise, adverse physiological changes are nevertheless taking place, with potentially serious consequences to human health.”

To further assess the noise-related disease burden, the working group estimated disability-adjusted life years (DALYs) due to noise-related CHD. DALYs reflect how much the expectancy of healthy life is reduced by premature death or by disability caused by disease. This measure lets policy makers compare disease burdens associated with different environmental factors and forecast the likely impact of preventive policies. The working group estimated that in 2002 Europeans lost 880,000 DALYs to CHD related to road traffic noise.

Chronic high levels of stress hormones such as cortisol, adrenaline, and noradrenaline can lead to hypertension, stroke, heart failure, and immune problems. According to a review of the research in the January–March 2004 issue of *Noise and Health*, arousal associated with nighttime noise exposure increased blood and saliva concentrations of these hormones even during sleep. “Taken together, recent epidemiologic data show us that noise is a major stressor that can influence health through the endocrine, immune, and cardiovascular systems,” says Prasher.

Other recent support for an association of cardiovascular mortality with noise comes from a study published in the 1 January 2007 issue of *Science of the Total Environment*. The results showed an 80% increased risk of cardiovascular mortality for women who judged themselves to be sensitive to noise. “Given these findings, noise sensitivity is a serious candidate to be a novel risk factor for cardiovascular mortality in women,” says Marja Heinonen-Guzejev, a research scientist at the University of Helsinki and lead author of the paper.

There is also a potential interaction between noise and air pollution, given that individuals exposed to traffic noise, for example, are often simultaneously exposed to air pollution. Prasher is currently investigating the effects of noise alone and in combination with chemical pollution.

The broader implications of chronic noise exposure also need to be considered. “Noise pollution contributes not only to cardiovascular disease, but also to hearing loss, sleep disruption, social handicaps, diminished productivity, impaired teaching and learning, absenteeism, increased drug use, and accidents,” says physician Louis Hagler, who coauthored a review on noise pollution in the March 2007 *Southern Medical Journal*. “The public health repercussions of increasing noise pollution for future generations could be immense.”

## Figures and Tables

**Figure:**